# Supplementary figures and images for: Persistent Disruptions in Prefrontal Connectivity Despite Behavioral Rescue by Environmental Enrichment in a Mouse Model of Rett Syndrome
Source: J Comp Neurol. 2025 Jul 17;533(7):e70073. doi: 10.1002/cne.70073 (PMC12269803; doi:10.1002/cne.70073)

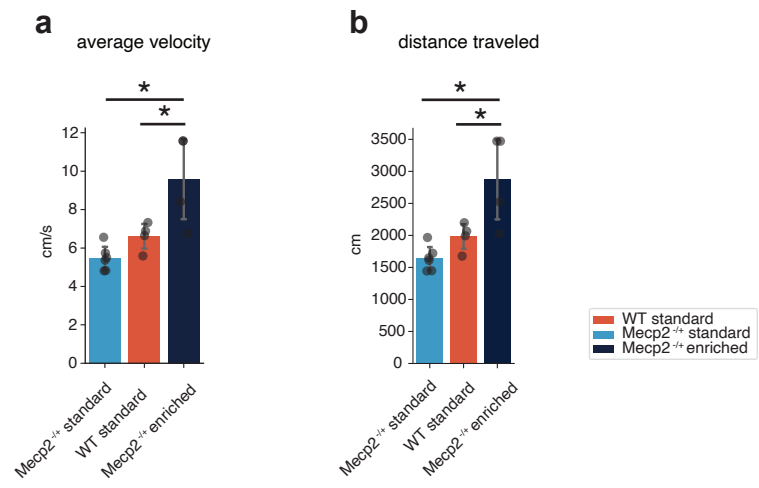

Supplement: Supplementary file 2 — Supplementary Figure: cne70073‐sup‐0002‐figureS1.pdf [file CNE-533-e70073-s005.pdf]

**a**

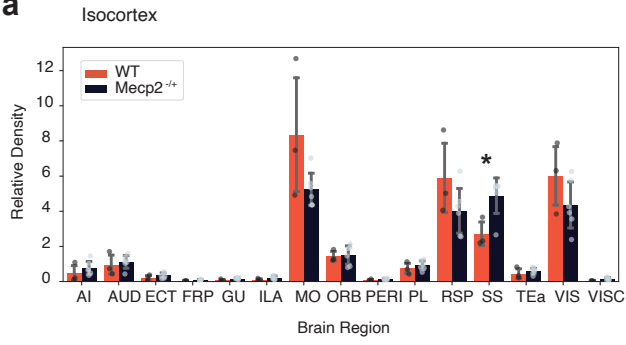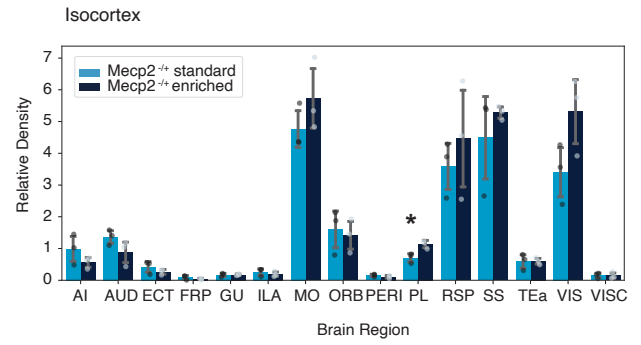

Supplement: Supplementary file 3 — Supplementary Figure: cne70073‐sup‐0003‐figureS2.pdf [file CNE-533-e70073-s003.pdf]

**a**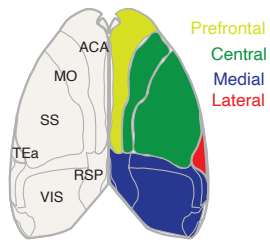**b**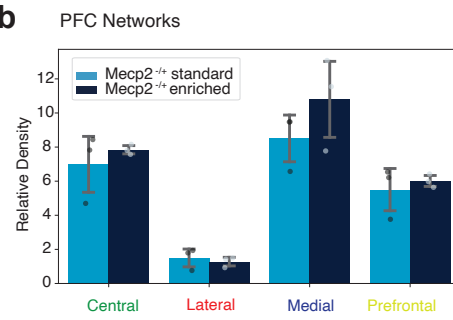**c**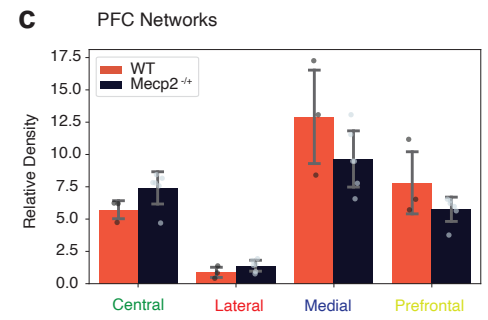**d**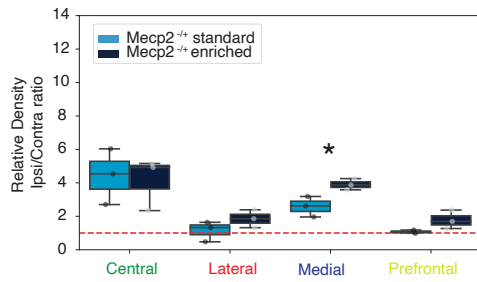**e**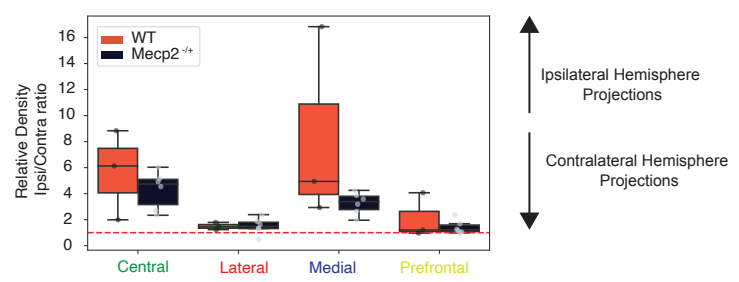

Supplement: Supplementary file 4 — Supplementary Figure: cne70073‐sup‐0004‐figureS3.pdf [file CNE-533-e70073-s001.pdf]

**a**      **Thalamus**

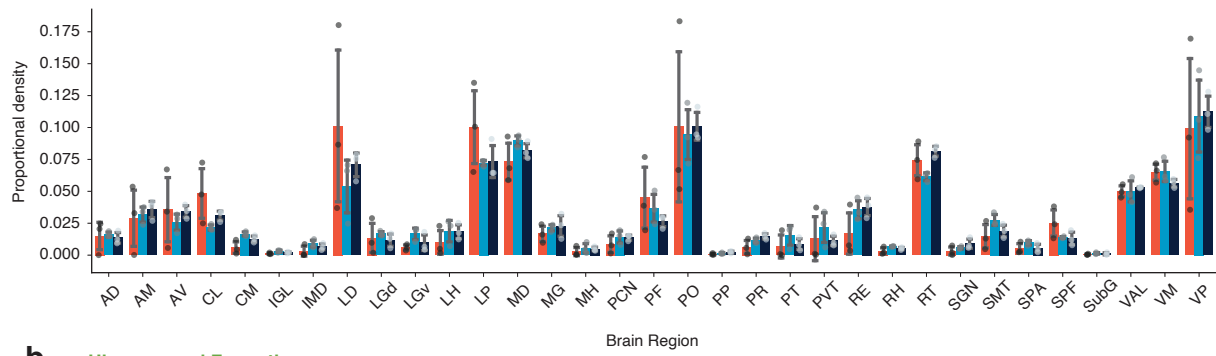

**b**      **Hippocampal Formation**

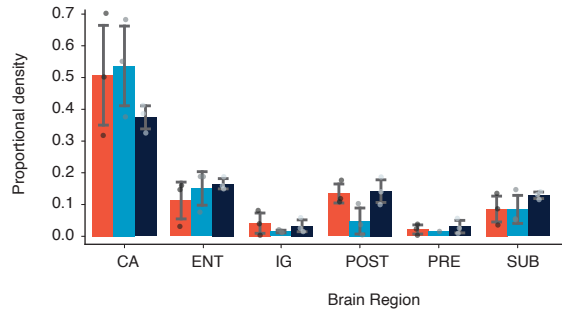

Supplement: Supplementary file 5 — Supplementary Figure: cne70073‐sup‐0005‐figureS4.pdf [file CNE-533-e70073-s002.pdf]
